# Temporal Dynamics of Socioeconomic Inequalities in COVID-19 Outcomes Over the Course of the Pandemic—A Scoping Review

**DOI:** 10.3389/ijph.2022.1605128

**Published:** 2022-08-29

**Authors:** Florian Beese, Julia Waldhauer, Lina Wollgast, Timo-Kolja Pförtner, Morten Wahrendorf, Sebastian Haller, Jens Hoebel, Benjamin Wachtler

**Affiliations:** ^1^ Division of Social Determinants of Health, Department of Epidemiology and Health Monitoring, Robert Koch Institute, Berlin, Germany; ^2^ Institute of Medical Sociology, Health Services Research and Rehabilitation Science, Faculty of Medicine and Faculty of Human Sciences, University of Cologne, Cologne, Germany; ^3^ Research Methods Division, Faculty of Human Sciences, University of Cologne, Cologne, Germany; ^4^ Institute of Medical Sociology, Centre for Health and Society (CHS), Medical Faculty, Heinrich-Heine University, Dusseldorf, Germany; ^5^ Division of Healthcare-Associated Infections, Surveillance of Antibiotic Resistance and Consumption, Department of Infectious Disease Epidemiology, Robert Koch Institute, Berlin, Germany

**Keywords:** COVID-19, health inequalities, pandemic preparedness, socioeconomic inequalities, temporal dynamics

## Abstract

**Objectives:** International evidence of socioeconomic inequalities in COVID-19 outcomes is extensive and growing, but less is known about the temporal dynamics of these inequalities over the course of the pandemic.

**Methods:** We systematically searched the Embase and Scopus databases. Additionally, several relevant journals and the reference lists of all included articles were hand-searched. This study follows the PRISMA guidelines for scoping reviews.

**Results:** Forty-six studies were included. Of all analyses, 91.4% showed stable or increasing socioeconomic inequalities in COVID-19 outcomes over the course of the pandemic, with socioeconomically disadvantaged populations being most affected. Furthermore, the study results showed temporal dynamics in socioeconomic inequalities in COVID-19, frequently initiated through higher COVID-19 incidence and mortality rates in better-off populations and subsequent crossover dynamics to higher rates in socioeconomically disadvantaged populations (41.9% of all analyses).

**Conclusion:** The identified temporal dynamics of socioeconomic inequalities in COVID-19 outcomes have relevant public health implications. Socioeconomic inequalities should be monitored over time to enable the adaption of prevention and interventions according to the social particularities of specific pandemic phases.

## Introduction

Since late 2019, the Severe Acute Respiratory Syndrome Corona Virus type 2 (SARS-CoV-2) and the corresponding coronavirus disease (COVID-19) have rapidly spread worldwide, leading to the declaration of COVID-19 as a pandemic in March 2020 by the World Health Organization (WHO) [1]. To date, the pandemic has led to approximately 397 million cumulative cases and 5.7 million cumulative deaths globally and remains a significant challenge for societies worldwide [2, 3].

Knowledge of the social epidemiological patterns in the distribution of SARS-CoV-2 infections and COVID-19 outcomes was limited at the beginning of the pandemic [4]. However, as soon as the first phases of the pandemic, several studies found socioeconomic inequalities in the risk of infection with SARS-CoV-2 and COVID-19 outcomes [4–7]. These early findings were confirmed by several international studies over the further course of the pandemic that presented additional evidence that socioeconomic inequalities in COVID-19 outcomes were observable in a variety of different national settings and at different time points during the pandemic [8].

But as with health inequalities in general [9], socioeconomic inequalities in COVID-19 outcomes may change or reproduce over time, leading to specific social epidemiological patterns of disease distribution during different phases of the pandemic. Studies from, e.g., Germany [10, 11] Hong Kong [12], and the United States [13] have described temporal dynamics from initially higher infection rates in more affluent populations and a later crossover to higher rates in socioeconomically disadvantaged populations. However, knowledge about specific temporal patterns of socioeconomic inequalities in COVID-19 remains limited and to date has not been systematically reviewed. Scientific evidence of temporal dynamics in the social epidemiological patterns of COVID-19 outcomes across populations will be vital to developing more targeted interventions and to guide future pandemic preparedness.

Using the example of the H1N1 pandemic in 2009–2010, Quinn and Kumar [14] emphasized the necessity of considering socioeconomic inequalities in general in pandemic preparedness plans. Because socioeconomically disadvantaged populations are generally at higher risk to get ill and have fewer resources to prevent infections or a severe course of the disease, an effective pandemic preparedness plan that addresses inequalities in exposure, susceptibility, and healthcare access is crucial to prevent or reduce an ongoing infectious trajectory, especially in today’s globalized world. Understanding the time-dependent patterns of socioeconomic inequalities in COVID-19 may therefore be helpful in identifying high-risk groups at different phases of the pandemic and inform targeted and timely public health interventions to reduce health inequalities and the overall burden of disease.

We therefore conducted a scoping review to map and synthesize the available evidence on temporal dynamics of socioeconomic inequalities in COVID-19 incidence, hospitalizations, and mortality.

## Methods

This scoping review follows the methodological steps described by Arksey and O’Malley [15], i.e., 1) identify the research question; 2) identify relevant literature; 3) study selection; 4) charting the data; and 5) collating, summarizing and reporting the results. We used the PCC mnemonic (Population, Concept, Context) to formulate the research question aiming to achieve comprehensive coverage of the available scientific literature [16]: What is known about the temporal dynamics of socioeconomic differences in COVID-19 incidence, hospitalizations, and mortality (Concept) during the pandemic (Context) in the context of high-income countries (Population)?

A study protocol was published in the Open Science Framework [17]. The present article follows the Preferred Reporting Items for Systematic Reviews and Meta-Analyses extension for Scoping Reviews (PRISMA-ScR, Supplementary Material S1) [18].

### Search Strategy

We conducted a systematic search in the electronic databases Embase and Scopus on 24 August 2021. The Embase database has predominantly indexed literature in the biomedical field, particularly in the fields of medicine, health sciences, and public health. In addition, Embase includes all records indexed in Medline since its coverage expansion in 2010 [19]. Scopus covers records in the fields of medicine, health sciences and economics, but has indexed references in the social sciences as well [20].

This review focused on the three following concepts: 1) SARS-CoV-2 and COVID-19, 2) socioeconomic inequalities, and 3) disease outcomes (incidence, hospitalizations, and mortality). We developed database-specific search strings using these three concepts. We excluded low- and middle-income countries according to the World Bank’s list published in 2021 [21]. For the search in Embase, the relevant terms of the Emtree thesaurus for each concept were included. The database-specific search strings are documented in the supplementary material (Supplementary Material S2).

In addition to the database search, we hand-searched a selection of peer-reviewed journals published by official national and regional public health institutions, i.e., the Journal of Health Monitoring and the Epidemiologisches Bulletin of the Robert Koch Institute, Germany, the Eurosurveillance journal of the European Centre for Disease Control and Prevention, and the Morbidity and Mortality Weekly Report and the Emerging Infectious Diseases journal of the Centers for Disease Control and Prevention. Furthermore, we iteratively identified the Public Library of Science and the International Journal of Environmental Research and Public Health during the database screening as most frequently publishing relevant content regarding the issue of temporal dynamics. We conducted the hand-search on 1 November 2021, covering publications from 1 January 2020 to 31 October 2021.

### Eligibility Criteria

We only included literature that met the eligibility criteria presented in Table 1. Studies were required to consider socioeconomic inequalities and temporal dynamics in COVID-19 incidence, hospitalizations, or mortality during the COVID-19 pandemic, i.e., to consider measures of corresponding health inequalities at least at two different time points. Eligible for inclusion were all empirical studies except for case studies, animal studies, pharmaceutical studies, and cell studies. Reviews were also eligible for inclusion if they were relevant in terms of the research question. Because scoping reviews do not necessarily aim to critically assess the quality of evidence of the included literature [15, 16], we included only peer-reviewed articles to assure inclusion of only articles that had at least some form of quality assessment.

**TABLE 1:** Eligibility criteria for the study selection (Germany, 2021).

	Inclusion	Exclusion
Study design	⁃ Repeated cross-sectional designs	⁃ Cross-sectional designs with one measure
	⁃ Cohort or longitudinal designs	⁃ Case studies
	⁃ Reviews	⁃ Animal studies
	⁃ Intervention studies	⁃ Cell studies
	⁃ Ecological studies	⁃ Pharmaceutical studies
	⁃ Case-control studies	
Publication type	⁃ Peer-reviewed articles	⁃ Conference contributions
	⁃ Peer-reviewed articles in press	⁃ Comments and scientific communications without presentation of own data analyses
		⁃ Essays
		⁃ Study preprints
		⁃ Study protocols
Populations	⁃ General population	⁃ Specified target populations with certain conditions (e. g. in-hospital patients)
Socioeconomic indicators	⁃ Income (and poverty)	⁃ Studies with an exclusive focus on race or ethnicity
	⁃ Education	
	⁃ Occupation	
	⁃ Employment	
	⁃ Composite measures (indices)	
Outcomes	⁃ Incidence (laboratory-confirmed)	⁃ Other outcomes
	⁃ Case counts	
	⁃ Mortality	
	⁃ Hospitalizations (number of hospital admissions)	
Regions/countries	⁃ High-income countries, according to the World Bank	⁃ Low-, lower-middle-, and upper-middle income countries, according to the World Bank
Languages	⁃ English or German	⁃ All other languages

This scoping review focused on studies that investigated the core dimensions of socioeconomic position, i.e., income, education, or occupation [22] as well as indices of socioeconomic deprivation and measures of employment measured at the individual or regional/national level. The population of interest refers to the general population on a national or regional level. We excluded low- and middle-income countries according to the classification of the World Bank [21] as the comparability of the significance of socioeconomic indicators and health outcomes might be very different due to the different contexts limiting the comparability [23]. We further excluded studies with rather homogenous study populations (e.g., specific occupational cohorts), as they do not allow for systematic comparisons of risks between socioeconomic groups [24].

### Study Selection and Data Extraction

Titles and abstracts of the retrieved records from the database search and journal hand-search, and subsequently the identified full texts, were independently screened by two reviewers (FB and LW). We hand-searched the reference lists of all included articles to identify additional eligible studies. We calculated the percent agreement and Cohen’s Kappa coefficient for both stages of study selection to determine the interrater reliability [25]. In case of conflicts, records were discussed within the research team until consent was reached. For computing Cohen’s Kappa statistic, we used R statistical software version 4.1.2 [26]. We systematically extracted relevant data on author and year, the title of the study, the country in which the study was conducted, research aims, methods and analytical approach, the underlying population, the observation period, and the outcome measures and socioeconomic variables that were analyzed, as well as relevant results and additional information.

### Data Synthesis and Presentation

A PRISMA flow chart was used to summarize and visualize the selection process [18]. To summarize the relevant evidence concerning temporal dynamics, we categorized the results as: 1) persistent, 2) growing, 3) decreasing, or 4) crossing over time, indicating a persistence of COVID-19 inequalities over time, growing or decreasing COVID-19 inequalities over time, or inequalities with crossover dynamics (e.g., from higher rates in COVID-19 outcomes in the more affluent to higher rates in less affluent populations over time), respectively. Furthermore, we categorized socioeconomic characteristics as income-based, education-based, occupation-based, employment-based, or index-based socioeconomic measures. We provide a summary table of the included studies covering author and year of publication, study location, COVID-19–related data, underlying populations or sample sizes, level (individual or ecological) and indicators of socioeconomic data, observation periods, outcome measures, and relevant results, i.e., the temporal pattern for the corresponding outcome and socioeconomic indicator. The principal descriptive findings were summarized graphically and numerically. Finally, these preliminary descriptive results were used to narratively synthesize the evidence [27].

## Results

**FIGURE 1:**
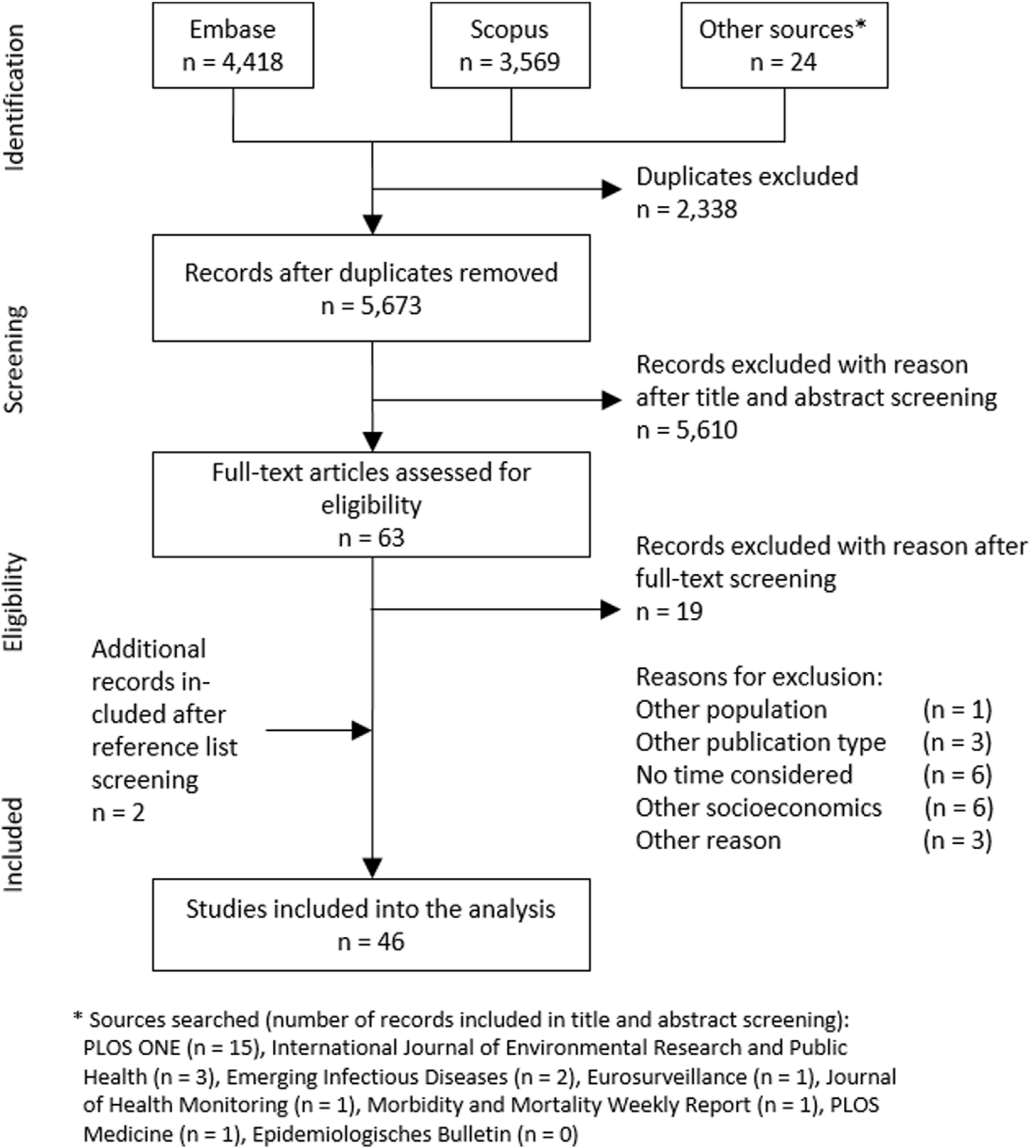
Flow chart of the study selection process, based on The Preferred Reporting Items for Systematic reviews and Meta-Analyses extension for Scoping Reviews (Australia, 2018).

We identified 46 full-text articles that met all eligibility criteria. Figure 1 shows a PRISMA flow chart with detailed information on the study selection process. We achieved an interrater percent agreement of 99.6% and a Cohen’s Kappa coefficient of 0.80 in the title and abstract screening. In the full-text screening, we achieved an interrater percent agreement of 88.9% and a Cohen’s Kappa coefficient of 0.71. The extracted data are presented for each study in a summary table (Table 2).

**TABLE 2:** Summary of findings (Germany, 2021).

First author (year)	Country	COVID data	Population, sample, cases, or area units, if applicable	Observation period and context if applicable	Socioeconomic indicators	Level of socioeconomic indicators	Outcome	Dynamic
[30]	ES	SD	357,989 participants	9 March 2020–13 December 2020 covering three Spanish pandemic waves	Index	E	Incidence	Crossover
			74,039 cases		Employment	I	Incidence	Crossover
			123 Basic Health Care Areas (BHA)		Income	I	Incidence	Persistent
[31]	PT	SD	42,523 cases	1 April 2020–1 July 2020 four cross-sections during and after lockdown	Index	E	Incidence	Crossover
			74 municipalities		Income	E	Incidence	Crossover
					Unemployment	E	Incidence	Persistent
[32]	US	SD	Population of Georgia, US	1 March 2020–31 August 2020 shelter-in-place order terminated at 1 May	Index	E	Incidence	Crossover
			159 US counties		Index	E	Mortality	Crossover
[33]	UK	SD	58,186 deaths	2 March 2020–3 December 2020	Index	E	Mortality	Growing
			32,844 lower-level super output areas					
[34]	IT	SD	36 provinces	24 February 2020–30 March 2020	Employment	E	Incidence	Growing
[35]	SE	SD	2,379,792 participants	9 March 2020–12 April 2020 refers to Swedish COVID-19 outbreak	Education	E	Mortality	Crossover
			1,942 deaths		Income	E	Mortality	Crossover
			5,984 Demographic Statistics Areas		Employment	E	Mortality	Crossover
[36]	US	SD	2,087 counties	1 March 2020–5 July 2020 SAH orders between March and April	Index	E	Incidence	Crossover
[13]	US	SD	3,141 US counties	22 January 2020–28 May 2020	Index	E	Incidence	Crossover
					Index	E	Mortality	Crossover
[37]	IT	SD	Population of Lombardy, IT 1,469 municipalities	1 January 2020–30 June 2020 Mortality peak in March	Income	E	Mortality	Persistent
[38]	IT	SD	32,588 cases	20 February 2020–3 May 2020	Education	E	Incidence	Growing
			2 provinces	Before and after lockdown	Unemployment	E	Incidence	Persistent
[39]	US	SD	3,142 US counties	8 March 2020–25 July 2020	Index	E	Incidence	Growing
[40]	NL	SD	2,700,563 tested individuals 99,412 cases	1 June 2020–17 October 2020 From September, priority testing for HCWs	Occupation	I	Incidence	Crossover
[41]	CH	SD	17,698 tested individuals 3,355 cases 2,830 Swiss Areas (SA) neighborhoods	26 February 2020–30 April 2020	Index	E	Incidence	Persistent
[28]	IT	CD	2,255 participants	7 May 2020–31 October 2020	Occupation	I	Incidence	Persistent
[42]	US	SD	3,142 US counties	15 March 2020–30 November 2020	Income	E	Incidence	Crossover
					Income	E	Mortality	Crossover
[43]	DE	SD	401 German districts	1 October 2020–15 December 2020 refers to second German wave	Index	E	Incidence	Crossover
					Index	E	Mortality	Crossover
[44]	DE	SD	401 German districts	3 February 2020–28 March 2021 covering two German waves and beginning of third wave	Income	E	Incidence	Crossover
					Employment	E	Incidence	Persistent
					Unemployment	E	Incidence	Crossover
[45]	US	SD	3,141 US counties	21 January 2020–30 June 2020 referring to first wave in US	Education	E	Incidence	Persistent
					Income	E	Incidence	Persistent
					Occupation	E	Incidence	Persistent
					Education	E	Mortality	Crossover
					Income	E	Mortality	Persistent
					Occupation	E	Mortality	Persistent
[46]	US	SD	2,853 US counties	21 January 2020–1 April 2020	Index	E	Incidence	Crossover
					Education	E	Incidence	Persistent
					Index	E	Mortality	Growing
					Education	E	Mortality	Persistent
[47]	UK	SD	417 Middle Level Super Output Areas	1 March 2020–31 May 2020	Income	E	Mortality	Persistent
					Occupation	E	Mortality	Persistent
					Education	E	Mortality	Persistent
[48]	US	SD	7 US states	3 May 2020–30 May 2020	Incidence	E	Index	Growing
[10]	DE	SD	401 German districts	31 August 2020–10 January 2021 referring to second German wave	Index	E	Incidence	Crossover
[49]	US	SD	3,092 US counties	22 January 2020–28 April 2020	Income	E	Incidence	Crossover
					Income	E	Mortality	Crossover
[50]	US	SD	3,123 US counties	1 April 2020–31 October 2020	Income	E	Mortality	Crossover
[51]	US	SD	4,289,283 cases	25 March 2020–29 July 2020	Index	E	Incidence	Growing
			147,074 deaths 3,137 US counties		Index	E	Mortality	Growing
[52]	US	SD	327,578 cases	2 March 2020–19 July 2020	Index	E	Incidence	Persistent
			7 US counties	Covering school closure date (16 March)				
[29]	US	RD	530 ZIP codes	1 January 2020–19 May 2020	Income	E	Mortality	Growing
[53]	US	SD	277,520 tested individuals	1 April 2020–30 April 2020	Income	E	Incidence	Growing
			124,135 cases					
			177 ZIP codes					
[54]	IL	SD	279 cities, towns, villages	31 March 2020–17 January 2021	Education	E	Incidence	Crossover
				Four cross-sections	Index	E	Incidence	Crossover
[55]	HK	SD	3,847 cases 18 geographic units of HK	23 January 2020–31 August 2020 Referring to three waves in HK	Index	E	Incidence	Crossover
[56]	US	SD	3,143 counties	22 January 2020–26 July 2020	Income	E	Incidence	Growing
					Income	E	Mortality	Growing
[57]	ES	SD	61,572 cases 1,068 area units	1 March 2020–30 November 2020	Index	E	Incidence	Growing
[58]	US	SD	3,142 US counties	15 March 2020–31 December 2020	Index	E	Incidence	Crossover
					Index	E	Mortality	Crossover
[59]	US	SD	2,664 US counties	1 April 2020–31 October 2020	Index	E	Incidence	Growing
				Two cross-sections	Index	E	Mortality	Growing
[60]	US	SD	316,626 tested individuals	1 March 2020–16 August 2020	Income	E	Incidence	Crossover
			37,546 cases		Education	E	Incidence	Persistent
			1,038 deaths		Education	E	Mortality	Persistent
			86 ZIP codes					
[61]	US	SD	3,108 US counties	1 April 2020–31 October 2020	Education	E	Mortality	Crossover
					Unemployment	E	Mortality	Crossover
[62]	DE	SD	401 German districts	14 April 2020–19 May 2020 Covering period of “hard lockdown” and period of relaxation	Unemployment	E	Incidence	Crossover
					Income	E	Incidence	Crossover
					Education	E	Incidence	Persistent
					Unemployment	E	Mortality	Crossover
					Income	E	Mortality	Crossover
					Education	E	Mortality	Persistent
[63]	DE	SD	401 German districts	10 June 2020–23 September 2020	Income	E	Incidence	Decreasing
[64]	UK	SD	3,456 hospitalizations	19 April 2020–15 September 2020	Index	E	Hospitalizations	Persistent
			11 Scotland mainland health boards	Six cross-sections				
[65]	UK	SD	774,491 tested individuals	1 March 2020–8 November 2020	Index	E	Incidence	Crossover
			75,173 cases		Index	E	Mortality	Persistent
			6,976 data zones		Index	E	Hospitalizations	Persistent
[66]	US	SD	28,306,349 cases	1 March 2020–28 February 2021 Covering early pandemic, late spring, summer, fall—school openings, winter—holiday session, winter—post-holiday travel	Index	E	Incidence	Crossover
			505,620 deaths 3,220 US counties		Index	E	Mortality	Crossover
[67]	US	SD	351 cities and towns in Massachusetts, US	2 March 2020–29 October 2020 Covering first wave, summer nadir, second wave	Education	E	Incidence	Crossover
					Occupation	E	Incidence	Persistent
[68]	US	SD	431 ZIP codes	6 April 2020–22 June 2020 Referring phase of widespread	Income	E	Incidence	Persistent
[11]	DE	SD	186,839 cases 401 German districts	15 March 2020–15 June 2020 Early pandemic	Index	E	Incidence	Crossover
[12]	HK	SD	4,811 cases 291 tertiary planning units	1 January 2020–31 August 2020 Referring three pandemic waves in HK	Education	E	Incidence	Crossover
					Income	E	Incidence	Crossover
					Occupation	E	Incidence	Crossover
					Education	I	Incidence	Crossover
					Income	I	Incidence	Crossover
					Occupation	I	Incidence	Crossover
[69]	US	SD	16,396 cases 199 US counties	1 March 2020–28 February 2021	Unemployment	E	Incidence	Growing
					Income	E	Incidence	Growing

DE, Germany; HK, Hong Kong; IL, Israel; IT, Italy; NL, Netherlands; PT, Portugal; ES, Spain; SE, Sweden; CH, Switzerland; UK, United Kingdom; US, United States; SD–surveillance data, CD, cohort data; RD, registry data; E—ecological, I—individual; SAH, stay-at-home; HCW, healthcare worker.

Most of the included studies were conducted in the United States (US, *n* = 23). The remaining studies were conducted in European countries (*n* = 20), such as Germany (*n* = 6), Italy (*n* = 4), and the United Kingdom (UK, *n* = 4), with three exceptions that were conducted in Hong Kong (*n* = 2) and Israel (*n* = 1). All of the included studies were observational; nine were published in 2020, while 37 were published in 2021. Most of the COVID-19-related data used in the included studies were derived from surveillance data (*n* = 44) and referred to notification data collected by public health institutions. One study used data from a seroepidemiological cohort study [28]. Another study used registry data on mortality [29].

### Socioeconomic Data

Socioeconomic data were mainly analyzed on an ecological level (*n* = 42). Two studies used individual socioeconomic data (*n* = 2), and two studies used both ecological and individual socioeconomic data (*n* = 2). Table 3 presents a detailed overview of socioeconomic indicators used across the included studies.

**Table 3:** Measure and number of socioeconomic indicators used by the included studies (Germany, 2021).

Measures of socioeconomic position	*n* ^a^	Studies
Individual	7	
Education level	1	[12]
Employment	1	[30]
Income	2	[12, 30]
Occupation categories	3	[12, 28, 40]
Ecological (area-based)	64	
Occupation- and employment-related	13	
% employed	3	[34, 35, 44]
% unemployed	6	[31, 38, 44, 61, 62, 69]
Occupation categories	4	[12, 45, 47, 67]
Education-related	11	
Mean education	1	[47]
% with primary, secondary, tertiary education	1	[12]
% educated above elementary school	1	[35]
% at most primary educated	1	[38]
% high school educated	1	[45]
% less then high school educated	1	[46]
% with matriculation certificate eligibility	1	[54]
% with at least college or university degree	4	[60–62, 67]
Income-related	18	
Average income	4	[31, 37, 62, 63]
Average household income	2	[47, 68]
Median income	4	[12, 35, 44, 69]
Median household income	4	[42, 53, 56, 60]
% living below the poverty line	4	[29, 45, 49, 50]
Indices	22	
Gini index of income inequality	2	[31, 66]
Indices of Multiple Deprivation	1	[33]
Area Deprivation Index	2	[36, 48]
Social Vulnerability Index	3	[39, 51, 58]
German Index of Socioeconomic Deprivation	2	[10, 11]
Scottish Index of Multiple Deprivation	2	[64, 65]
Other indices	10	[13, 30, 32, 41, 46, 52, 54, 55, 57, 59]

a Refers to the number of analyses conducted within the included studies using the corresponding indicator.

### Outcomes Measures

COVID-19 incidence was the most researched outcome across the included studies (*n* = 38). COVID-19 incidence was predominantly measured by notification data that referred to laboratory-confirmed cases of SARS-CoV-2 infection. Three studies on COVID-19 incidence described case increments over a certain time period [34, 51, 59]. Some studies created other outcomes based on incidence measures [28, 39, 41, 45]. For instance, Dasgupta et al. [39] investigated the association of socioeconomic variables with the risk of becoming a hotspot across US counties based on incidence rates.

Two studies researched COVID-19 hospitalizations over time using governmental data on hospitalizations and primary care data from national healthcare records [64, 65].

Mortality was the second-most researched outcome across the included studies (*n* = 22). While most of the studies used confirmed deaths related to COVID-19, three studies analyzed excess mortality by comparing mortality data from recent years with time periods during the pandemic [29, 35, 37]. Two studies investigated death increments over time [51, 59]. One study used case fatality rates [32].

### Observation Periods

The earliest start date was 1 January 2020 [12, 29, 37], and the latest start date was 1 October 2020 [43]. The mean observation period across the included studies was 23.4 weeks, with a median period of 19.9 weeks and a period range of 3.9–59.9 weeks. Most of the studies used surveillance data and analyzed temporal dynamics according to daily, weekly, or monthly outcome rates. However, several studies compared two or more waves or several time points referring to certain contexts, such as the implementation of stay-at-home orders. Supplementary Material S3 shows the observation periods of the included studies.

### Temporal Dynamics

The included studies conducted a total of 93 analyses that combined different socioeconomic indicators with COVID-19 outcomes. Of those, 51.6% (*n* = 48) found crossover dynamics in socioeconomic inequalities, 29% (*n* = 27) found persistent inequalities, 18.3% (*n* = 17) found growing inequalities, and 1.1% (*n* = 1) found decreasing inequalities over time. Among the 48 analyses concluding crossover dynamics in socioeconomic inequalities, 81.3% (*n* = 39) showed crossover dynamics over time from initially higher outcome rates in better-off populations to higher rates in more deprived populations. Most of the analyses (91.4%, *n* = 85) showed constant or growing socioeconomic inequalities in COVID-19 outcomes, with socioeconomically disadvantaged populations being most affected.

### Dynamics of Socioeconomic Inequalities in COVID-19 Incidence

**FIGURE 2:**
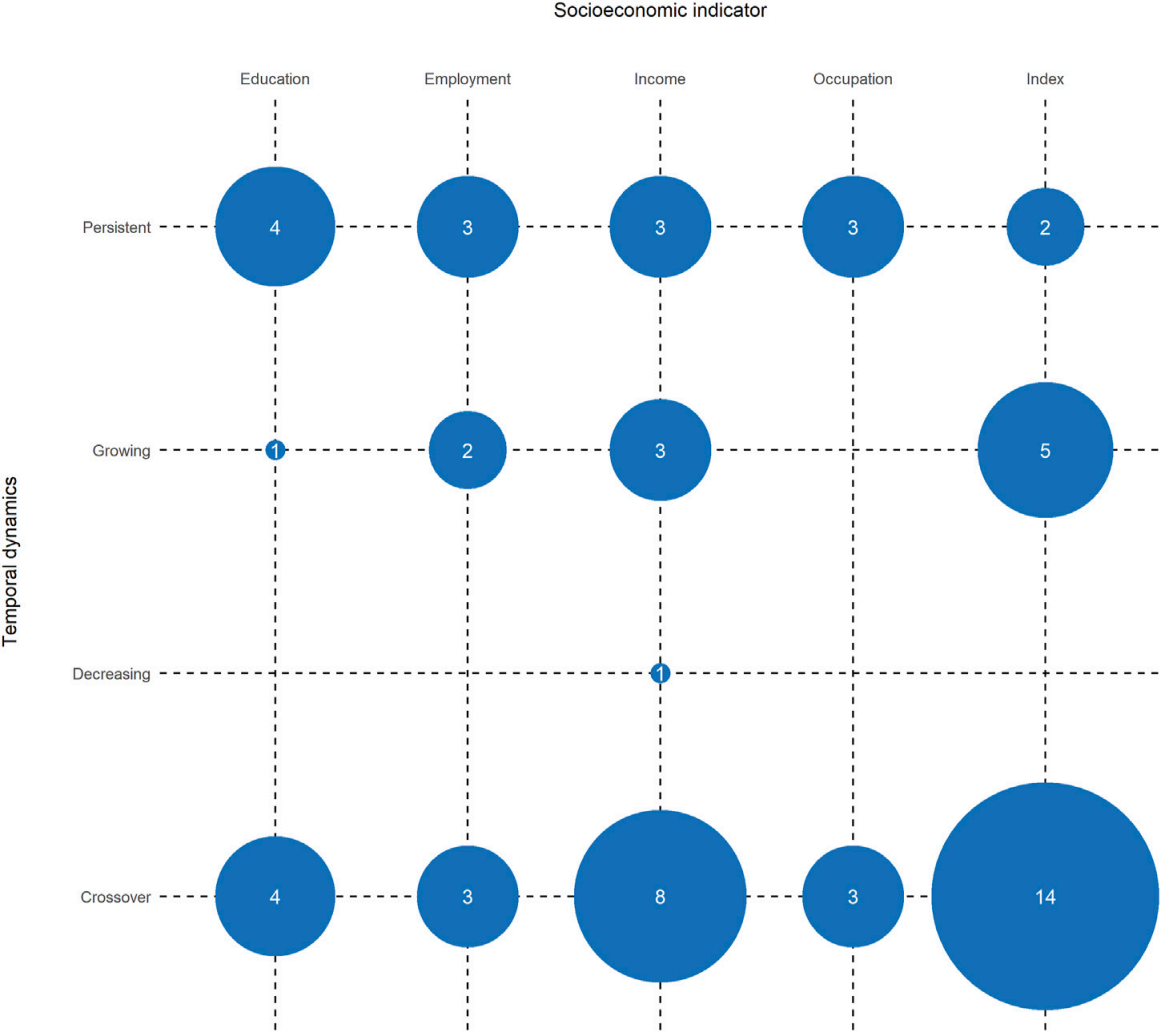
Number of included analyses that found particular temporal dynamics of socioeconomic inequalities in COVID-19 incidence (Germany, 2021).

Figure 2 shows the temporal dynamics of socioeconomic inequalities in COVID-19 incidence by socioeconomic indicators. Similar to the proportions across all analyses, of the analyses of incidence rates (*n* = 59), 54.2% (*n* = 32) found crossover dynamics in socioeconomic inequalities, 25.4% (*n* = 15) found persistent inequalities, 18.6% (*n* = 11) found growing inequalities, and 1.7% (*n* = 1) found decreasing inequalities over time.

Several studies examined the association of area-based indices of socioeconomic deprivation with incidence rates and found crossover dynamics over time. For instance, Aguilar-Palacio et al. investigated the impact of the Basic Health Care Deprivation Index on COVID-19 incidence across three infection waves in Aragón, Spain [30]. They concluded that incidences were higher in more affluent populations in the first wave of the pandemic but inverted with ongoing progression, manifesting in higher rates in socioeconomically disadvantaged populations in waves 2 and 3. Chang et al., Neelon et al., and Clouston et al. investigated infection rates in the US and found similar results at the county level [13, 36, 58]. Furthermore, Neelon et al. showed sinusoidally shaped temporal dynamics of socioeconomic inequalities in COVID-19 incidence, indicating several crossovers over time [58] similar to one study from the UK [65]. Wachtler et al. used the German Index of Socioeconomic Deprivation to explain COVID-19 incidence very early in the pandemic across 401 German districts, concluding that the rates were initially higher in more affluent districts but tended to cross over to higher rates in more deprived districts already during the first pandemic wave [11]. Hoebel et al. found similar patterns at the district level during the second pandemic wave in Germany [10]. However, Doblhammer et al. used a different, machine-learning–based approach to identify socioeconomic indicators combined into composite measures and their impact on COVID-19 cases at the district level during the second pandemic wave in Germany [43]. Although the analytical approach differed from that of Hoebel et al., the authors found similar patterns, indicating that less deprived areas were more strongly correlated with cases at the beginning of the second wave but the correlation later reversed to the detriment of more deprived areas.

Yang et al. investigated incidence rates with socioeconomic indicators on both the ecological and individual levels in Hong Kong and found similar crossover dynamics with initially higher rates in more affluent travelers and students [12]. Crossover dynamics were also present in multiple studies concerning income [12, 31, 42, 44, 49, 60, 62], education [12, 54, 67], and employment [30, 44, 62].

Karmakar et al. investigated the association of an area-based index with COVID-19 incidence in US counties [51]. The results show that growth rates in more deprived counties were steeper than in more affluent counties, indicating growing inequalities in the distribution of COVID-19 cases. Similar results were found by other studies [48, 57, 59]. Dasgupta et al. researched the impact of area-based socioeconomic vulnerability on emerging hotspots across US counties [39]. In general, more deprived counties were at higher risk of becoming a hotspot; once a hotspot appeared, the temporal increase of cases was steeper with higher deprivation. Growing inequalities in COVID-19 incidence rates were also described in analyses concerning income [53, 56, 69], education [38], or employment [69].

### Dynamics of Socioeconomic Inequalities in COVID-19 Hospitalizations

Studies that considered hospitalizations (*n* = 2) found persistent inequalities to the disadvantage of socioeconomically disadvantaged populations. While Rideout et al. did not find statistically significant socioeconomic inequalities in hospitalization rates, Simpson et al. identified a higher risk for socioeconomically disadvantaged populations at all time points during the observation period [64, 65].

### Dynamics of Socioeconomic Inequalities in COVID-19 Mortality

**FIGURE 3:**
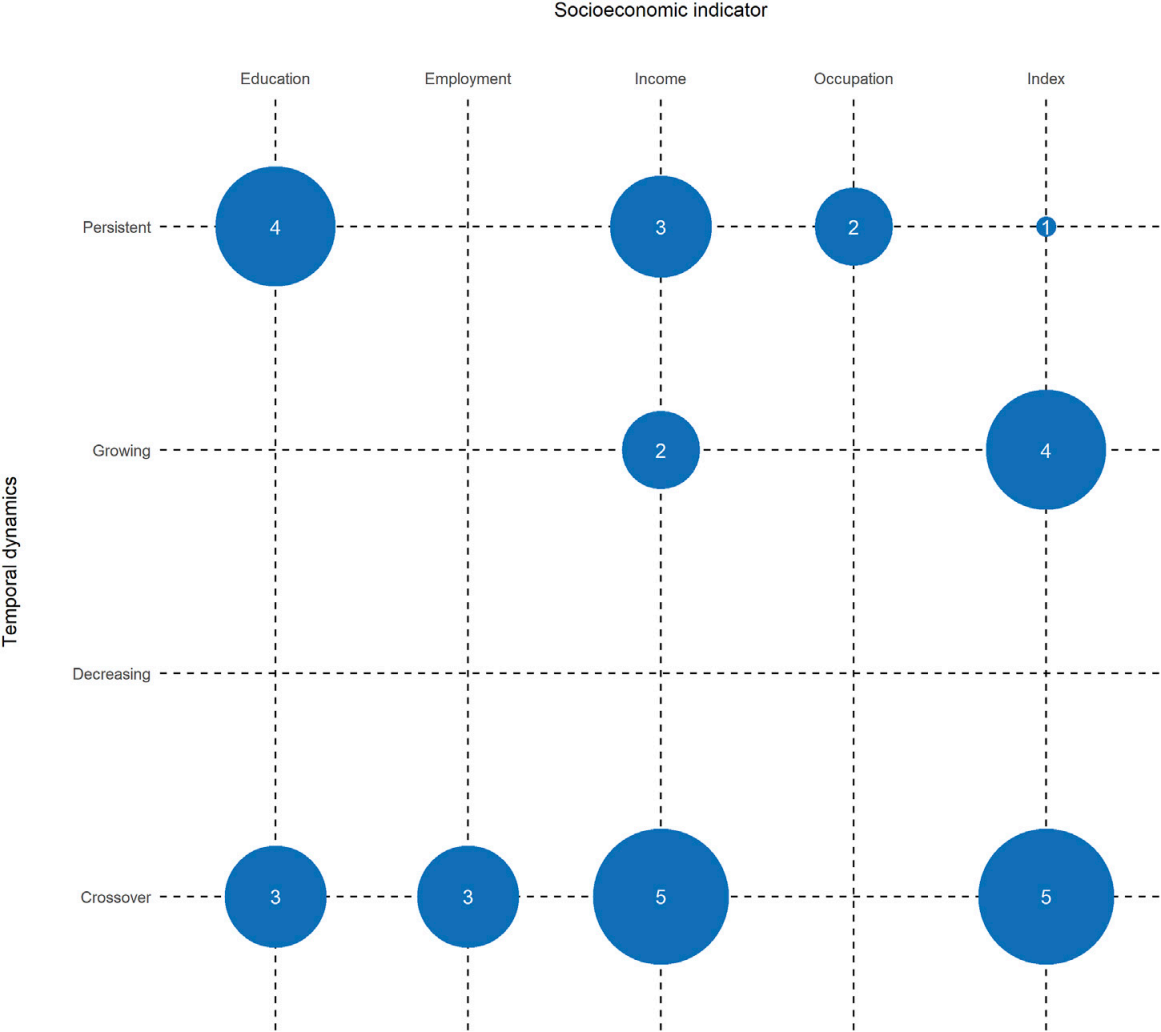
Number of included analyses that found particular temporal dynamics of socioeconomic inequalities in COVID-19 mortality (Germany, 2021).

Figure 3 shows the temporal dynamics of socioeconomic inequalities in COVID-19 mortality by socioeconomic indicators. Among the analyses investigating mortality rates (*n* = 32), we found results similar to those for COVID-19 incidence rates (crossover: 50%, *n* = 16; persistent: 31.3%, *n* = 10; growing: 18.8%, *n* = 6). For instance, Clouston et al., Neelon et al., and Doblhammer et al. found that more affluent regions had higher mortality rates at the beginning of the respective observation periods, while at later time periods, the rates were elevated in more deprived regions [13, 43, 58]. Those dynamics were also present in other study results concerning income [35, 42, 49, 50, 62], education [35, 45, 61], or employment [35, 61, 62].

Other studies concluded that inequalities in COVID-19 mortality increased over time. For instance, Brown et al. and Karmakar et al. investigated the association of area-based socioeconomic indices with mortality rates in the UK and US, respectively [33, 51]. Finch et al. analyzed the impact of a poverty index on COVID-19 mortality in US counties [46]. All of these studies concluded that index-related inequalities in COVID-19 mortality increased over time, with generally higher mortality rates in the most deprived region. Growing inequalities concerning income were also present [29]. Maiti et al. did not provide a direction of the association due to their analytical approach exploring the impact of ecological socioeconomic variables in all US counties on explaining COVID-19 mortality; instead, they concluded that the impact of socioeconomic factors such as median household income on explaining COVID-19 mortality increased over time [56]. When investigating the association of occupational indicators and COVID-19 mortality, the included studies found persistent inequalities [45, 47]. Analyses of educational differences in COVID-19 mortality also found persistent inequalities to be present [46, 47, 60, 62].

## Discussion

This study shows that patterns of initially higher COVID-19 outcome rates in more affluent populations and subsequent crossover dynamics to higher COVID-19 outcome rates in the more deprived populations are internationally present in high-income countries. Moreover, several study results show that crossover dynamics frequently occur over time leading to opposing sinusoidal patterns. The results indicate that well-described inequalities in health appear to have persisted or even increased throughout the pandemic, predominantly to the disadvantage of more deprived populations. However, analyses to estimate the effect of socioeconomic factors on the overall burden of COVID-19 outcomes, such as the population attributable fraction, are still lacking but would be highly desirable for future research.

The included studies presented different possible explanations for the observed temporal patterns. Particularly for COVID-19 incidence and infection rates, the studies seem to confirm the important impact of mobility and the possibility of reducing mobility when advised by public health authorities. Doblhammer et al. discussed that mobility was higher in low infection periods among the more affluent populations than among less affluent populations [43]. This might have economic and occupational reasons: more affluent populations have a higher proportion of individuals working in jobs involving business travel, which may have introduced the virus early in the pandemic [11, 12, 42, 55, 62]. Higher incomes also provide the financial resources to travel during holidays, increasing the risk of introducing the virus into defined and socioeconomically more homogeneous local populations [62]. This would be in line with findings such as those from Germany, where no socioeconomic inequalities in infections were found in well-contained early hotspots [70]. Furthermore, this would be in line with the conceptualization of temporal dynamics in disease distribution proposed by Clouston et al. [71] using stages of disease theory. In the first stage, individuals have biologically the same risk of getting infected. In this stage, socioeconomic differences in disease distribution may occur at random. However, as Bambra conceptualized paths of socioeconomic inequalities in emerging infectious diseases, unequal exposures due to different sets of resources and conditions may contribute to socioeconomic inequalities in health and disease [72, 73]. In the case of the early COVID-19 pandemic, better-off populations may have been at higher risk due to their higher mobility.

However, when mobility restrictions were implemented, populations in lower-paid occupations, precarious jobs, and those living in higher-density housing or using public transport may have been less able to maintain physical distancing, while better-off populations tend to have more resources to follow infection protection restrictions, such as the ability to work remotely [13, 36, 42–44, 49, 60, 66, 74]. Furthermore, populations of lower education may have had insufficient information about the pandemic situation. Relevant information or preventive guidance may not have met the requirements of certain living environments of lower-educated or socioeconomically deprived populations, therefore leading to reduced compliance with recommended mitigation strategies [38, 44]. This would be in line with Bambra’s conceptualization of unequal transmission across different socioeconomic groups as it implies “inequality in the passing of a pathogen between community members” [72]. According to Clouston et al., socioeconomic inequalities arise during the second stage of disease [71]. Some populations will fare better with new prevention strategies and treatments, while others will face difficulties in prevention and treatment of emerging diseases due to strained resources or their living environment.

In addition, these inequalities might also be linked to social identity processes. Pre-existing social identities of individuals (such as living in deprived areas, working in essential or precarious jobs) and the belief of collectively being more affected due to the pandemic situation might lead to the emergence of new social group identities that might impact individuals’ behavior [75, 76]. For instance, in the US and Europe, right-wing populists instrumentalized public health measures such as mask-wearing to declare new social identities, promoting the social distinction between “us” and “them,” “encourage citizens to risk their health” [77] and reducing the compliance of certain communities with mitigation strategies. This might as well have influenced the time depending socioeconomic inequalities in the distribution of COVID-19, provided that these identities were associated with the individuals’ socioeconomic positions.

The majority of the included studies recommended public health measures that are relevant for future pandemic preparedness, such as improved low-threshold public health communication [39, 45, 51], coordination of community-based social services that meets the needs of local communities [51], promoting equity in testing capabilities [46, 52] and vaccine distribution [13, 32, 38, 45], providing personal protective equipment [66] or generally speaking, the implementation of specifically tailored interventions for socially disadvantaged populations by investing more resources [10, 11, 28, 30, 31, 35–39, 41, 44, 49, 50, 53, 54, 60, 61, 66]. Furthermore, some studies found, that the identification of vulnerable groups and data on specific subgroups were needed to enable targeted support [30, 31, 35, 40, 44, 48, 51, 60, 67].

Similar patterns of socioeconomic inequalities were observed for COVID-19 mortality as well across the included studies. A possible explanation for higher mortality rates in socioeconomically disadvantaged populations could be the well-described higher prevalence of chronic health conditions that are risk factors for severe and fatal COVID-19 in populations with a lower socioeconomic level. This leads to unequal susceptibility as described by Bambra [72]. Furthermore, populations with a lower socioeconomic level usually face greater barriers in accessing healthcare or even have geographical barriers, especially in rural regions [13, 32, 33, 35, 37, 49, 51]. This unequal treatment as conceptualized by Bambra might lead to a higher risk of facing a severe COVID-19 trajectory or even death for socioeconomically disadvantaged populations [72]. Low-threshold accessibility to diagnosis, treatment and healthcare should be provided to face this issue, as recommended by two of the included studies [30, 51].

This is the first systematically conducted review on the temporal dynamics of socioeconomic inequalities in COVID-19 outcomes. The systematic literature search contributes to a high comprehensiveness and assures reproducibility of the results, and the Embase and Scopus databases provide broad coverage of literature in the biomedical and social science fields. However, we cannot completely rule out that searches in additional databases could have yielded additional relevant records. We attempted to minimize this possibility by hand-searching relevant journals and the reference lists of all included studies in addition to the database searches.

Eligible studies were restricted to those conducted in high-income countries. Thus, the generalizability of the presented results to the contexts of low- and middle-income countries is limited. However, we also identified two studies from Brazil that ecologically analyzed social determinants of COVID-19 outcomes. Both studies concluded similar temporal patterns as most of the included studies, indicating that outcome rates initially were higher in more affluent populations but shifted to a higher burden in poorer populations over the course of the pandemic [78, 79].

Due to the pronounced heterogeneity of the included studies, no structured quality-of-evidence assessment could be conducted. This could potentially have led to biased results in the narrative synthesis as methodical limitations of the studies were not systematically assessed and considered [80]. This approach, however, seemed to be most appropriate given the aims of the review, i.e., to systematically generate a comprehensive overview of the international evidence on temporal dynamics in COVID-19 outcomes across different socioeconomic populations. Furthermore, the included studies were very heterogenous regarding socioeconomic indicators, observation periods and reported effect estimates. We therefore were not able to conduct any further quantitative analyses or systematical comparisons of effect estimates but instead summarized them narratively. Whereas this is often the case in scoping reviews as a result of the relatively broad research question, it limits our results to a qualitative interpretation. Further research is needed to quantitively describe the magnitude of the association between socioeconomic indicators and COVID-19 outcomes over time. To answer this research question, future reviews will have to focus on smaller sets of socioeconomic indicators and outcomes.

Publication bias is a potential issue because this review focused exclusively on peer-reviewed publications [81]. This bias could possibly have been minimized by including non-peer-reviewed literature and so-called grey literature. However, because no structured quality-of-evidence assessment could be conducted, eligibility was restricted to peer-reviewed articles to increase the validity of the included study results.

Our review may help to inform pandemic preparedness and emphasizes that social determinants of health, such as living and working conditions, provide relevant entry points for infection protection and control during epidemics with novel respiratory pathogens. As socioeconomic inequalities can be seen as unrealized opportunities to increase population health and health equity, targeted and timely prevention and intervention programs considering a population’s socioeconomic heterogeneity and the changing patterns of socioeconomic inequalities should be part of future pandemic preparedness plans. Considering the temporal dynamics may help to minimize the detrimental effects on specific groups during certain pandemic phases and should help to inform more specific containment measures. Public health interventions such as increased testing capabilities, vaccination programs, information campaigns, and healthcare access should meet the requirements of socioeconomically diverse populations—during the current COVID-19 pandemic and beyond—to strengthen the overall resilience of societies against new emerging infectious diseases and to tackle health inequities.
