# Supplementary figures and images for: Temporal Dynamics of Socioeconomic Inequalities in COVID-19 Outcomes Over the Course of the Pandemic—A Scoping Review
Source: Int J Public Health. 2022 Aug 29;67:1605128. doi: 10.3389/ijph.2022.1605128 (PMC9464808; doi:10.3389/ijph.2022.1605128)

**Supplementary Material S3.** Study durations of the included studies.


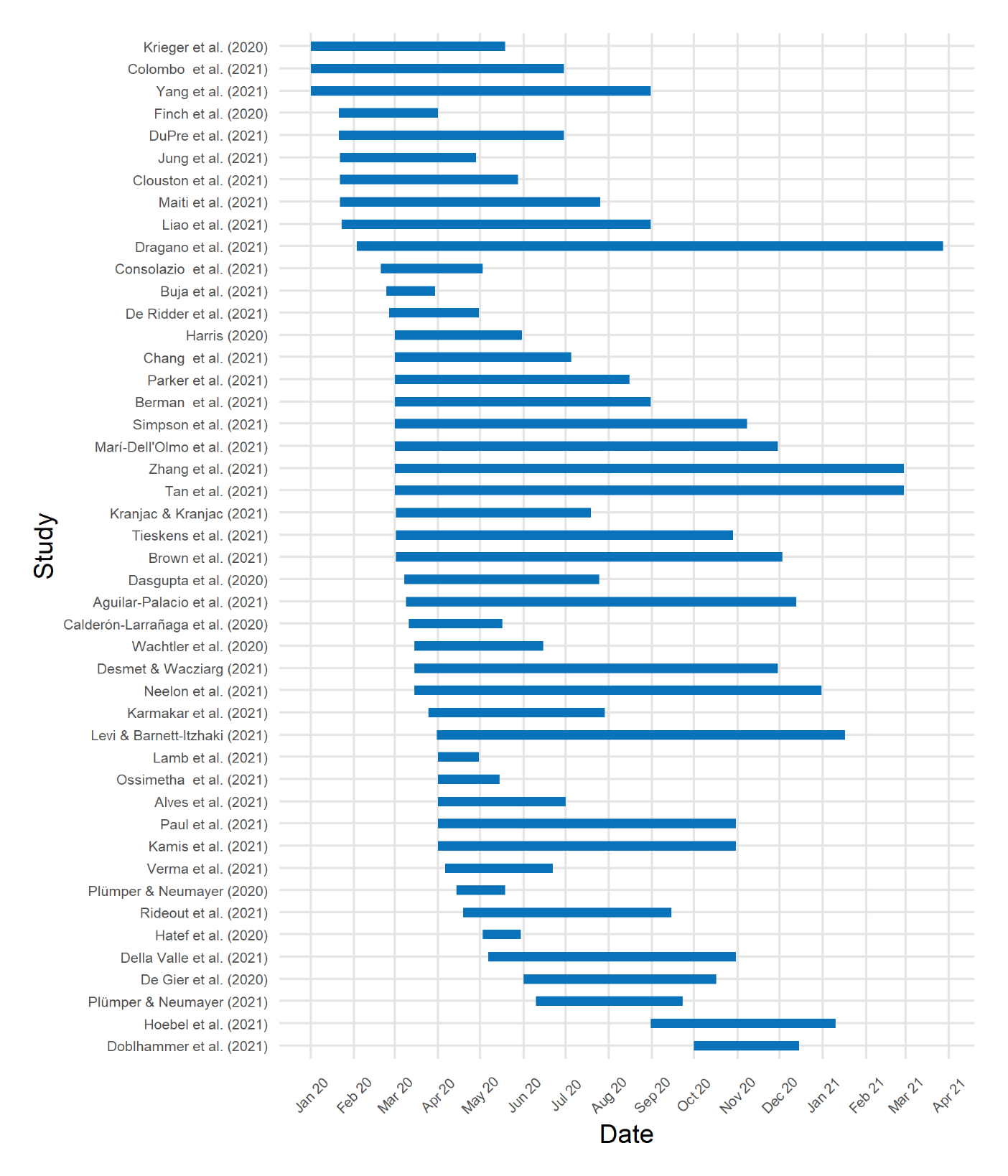

Supplement: Supplementary file 1 [file DataSheet3.docx]
