# Supplementary material for: Temporal Dynamics of Socioeconomic Inequalities in COVID-19 Outcomes Over the Course of the Pandemic—A Scoping Review
Source: Int J Public Health. 2022 Aug 29;67:1605128. doi: 10.3389/ijph.2022.1605128 (PMC9464808; doi:10.3389/ijph.2022.1605128)
Supplement: Supplementary file 2 [file DataSheet2.docx]

**Supplementary Material S2.** Database-specific search strings

**Embase**

('severe acute respiratory syndrome coronavirus 2':ab,ti OR 'covid 19':ab,ti OR ncov*:ab,ti OR covid*:ab,ti OR 'sars cov 2':ab,ti OR 'sars-cov-2':ab,ti OR 'sars coronavirus 2':ab,ti OR 'severe acute respiratory syndrome cov 2':ab,ti OR 'wuhan coronavirus':ab,ti OR 'wuhan seafood market pneumonia virus':ab,ti OR sars2:ab,ti OR '2019-ncov':ab,ti OR 'hcov-19':ab,ti OR 'novel 2019 coronavirus':ab,ti OR '2019 novel coronavirus*':ab,ti OR 'novel coronavirus 2019':ab,ti OR '2019 novel human coronavirus*':ab,ti OR 'human coronavirus 2019':ab,ti OR 'coronavirus disease-19':ab,ti OR 'corona virus disease-19':ab,ti OR 'coronavirus disease 2019':ab,ti OR 'corona virus disease 2019':ab,ti OR '2019 coronavirus disease':ab,ti OR 'novel coronavirus 2019*':ab,ti OR 'novel coronavirus disease 2019':ab,ti OR 'novel coronavirus infection 2019':ab,ti OR '2019 corona virus disease':ab,ti OR 'new coronavirus*':ab,ti OR 'coronavirus outbreak':ab,ti OR 'coronavirus epidemic':ab,ti OR 'coronavirus pandemic':ab,ti OR 'pandemic of coronavirus':ab,ti OR 'coronavirus disease 2019'/exp OR 'severe acute respiratory syndrome coronavirus 2'/exp)

AND

('socioeconomics':ab,ti OR socioeconomics:ab,ti OR 'socioeconomic status':ab,ti OR 'socio-economic status':ab,ti OR 'socioeconomic position':ab,ti OR 'socio-economic position':ab,ti OR 'social determinants':ab,ti OR 'socioeconomic determinants':ab,ti OR 'socio-economic determinants':ab,ti OR 'social status':ab,ti OR 'social position':ab,ti OR 'socioeconomic class':ab,ti OR 'socio-economic class':ab,ti OR 'social class':ab,ti OR 'health disparities':ab,ti OR 'health inequalities':ab,ti OR 'social disparities':ab,ti OR 'social inequalities':ab,ti OR 'socioeconomic disparities':ab,ti OR 'socioeconomic inequalities':ab,ti OR 'socio-economic disparities':ab,ti OR 'socio-economic inequalities':ab,ti OR 'social gradient':ab,ti OR 'socioeconomic gradient':ab,ti OR 'socio-economic gradient':ab,ti OR 'socioeconomic differences':ab,ti OR 'socio-economic differences':ab,ti OR 'social differences':ab,ti OR 'health differences':ab,ti OR 'ses':ab,ti OR 'sep':ab,ti OR income:ab,ti OR 'income inequality':ab,ti OR education:ab,ti OR 'educational status':ab,ti OR 'educational inequality':ab,ti OR employment:ab,ti OR 'employment status':ab,ti OR occupation:ab,ti OR occupational:ab,ti OR 'wealth*':ab,ti OR 'socioeconomics'/exp OR 'social status'/exp)

AND

(prevalence:ab,ti OR 'incidence':ab,ti OR incidence:ab,ti OR 'cumulative incidence':ab,ti OR 'incidence density rate':ab,ti OR 'incidence-density-rate':ab,ti OR 'person-time incidence rate':ab,ti OR 'attack rate':ab,ti OR 'attack-rate':ab,ti OR 'case rate':ab,ti OR 'case-rate':ab,ti OR 'case notification rate':ab,ti OR 'case-notification-rate':ab,ti OR 'infection risk':ab,ti OR 'infection-risk':ab,ti OR 'infection':ab,ti OR infection:ab,ti OR 'mortality':ab,ti OR mortality:ab,ti OR 'mortality-rate':ab,ti OR 'death rate':ab,ti OR 'death-rate':ab,ti OR 'death*':ab,ti OR hospitalisation:ab,ti OR 'hospitalisation rate':ab,ti OR 'hospitalisation-rate':ab,ti OR 'hospitalization':ab,ti OR hospitalization:ab,ti OR 'hospitalization rate':ab,ti OR 'hospitalization-rate':ab,ti OR 'mortality rate'/exp)

AND

([english]/lim OR [german]/lim)

AND

[2020-2021]/py

NOT

(afghanistan:ab,ti OR 'burkina faso':ab,ti OR burundi:ab,ti OR 'central african republic':ab,ti OR chad:ab,ti OR eritrea:ab,ti OR ethiopia:ab,ti OR gambia:ab,ti OR guinea:ab,ti OR 'guinea-bissau':ab,ti OR 'north korea':ab,ti OR liberia:ab,ti OR madagascar:ab,ti OR malawi:ab,ti OR mali:ab,ti OR mozambique:ab,ti OR niger:ab,ti OR rwanda:ab,ti OR 'sierra leone':ab,ti OR somalia:ab,ti OR 'south sudan':ab,ti OR sudan:ab,ti OR 'syrian arab republic':ab,ti OR syria:ab,ti OR togo:ab,ti OR uganda:ab,ti OR yemen:ab,ti OR angola:ab,ti OR algeria:ab,ti OR bangladesh:ab,ti OR belize:ab,ti OR benin:ab,ti OR bhutan:ab,ti OR bolivia:ab,ti OR 'capo verde':ab,ti OR cambodia:ab,ti OR cameroon:ab,ti OR comoros:ab,ti OR congo:ab,ti OR 'cote d`ivoire':ab,ti OR djibouti:ab,ti OR egypt:ab,ti OR 'el salvador':ab,ti OR eswatini:ab,ti OR ghana:ab,ti OR haiti:ab,ti OR honduras:ab,ti OR indonesia:ab,ti OR iran:ab,ti OR kenya:ab,ti OR kiribati:ab,ti OR kyrgyzstan:ab,ti OR laos:ab,ti OR lesotho:ab,ti OR mauritania:ab,ti OR 'federated states of micronesia':ab,ti OR mongolia:ab,ti OR morocco:ab,ti OR myanmar:ab,ti OR nepal:ab,ti OR nicaragua:ab,ti OR nigeria:ab,ti OR pakistan:ab,ti OR 'papua new guinea':ab,ti OR philippines:ab,ti OR samoa:ab,ti OR 'sao tome':ab,ti OR principe:ab,ti OR senegal:ab,ti OR 'solomon islands':ab,ti OR 'sri lanka':ab,ti OR tanzania:ab,ti OR tajikistan:ab,ti OR 'timor leste':ab,ti OR tunisia:ab,ti OR ukraine:ab,ti OR uzbekistan:ab,ti OR vanuatu:ab,ti OR vietnam:ab,ti OR 'gaza strip palestine':ab,ti OR zambia:ab,ti OR zimbabwe:ab,ti OR albania:ab,ti OR 'american samoa':ab,ti OR argentina:ab,ti OR armenia:ab,ti OR azerbaijan:ab,ti OR belarus:ab,ti OR bosnia:ab,ti OR herzegovina:ab,ti OR botswana:ab,ti OR bulgaria:ab,ti OR colombia:ab,ti OR 'costa rica':ab,ti OR cuba:ab,ti OR dominica:ab,ti OR 'dominican republic':ab,ti OR 'equatorial guinea':ab,ti OR ecuador:ab,ti OR fiji:ab,ti OR gabon:ab,ti OR grenada:ab,ti OR guatemala:ab,ti OR guyana:ab,ti OR iraq:ab,ti OR jamaica:ab,ti OR jordan:ab,ti OR kazakhstan:ab,ti OR kosovo:ab,ti OR lebanon:ab,ti OR libya:ab,ti OR malaysia:ab,ti OR maldives:ab,ti OR 'marshall islands':ab,ti OR mauritius:ab,ti OR mexico:ab,ti OR moldova:ab,ti OR montenegro:ab,ti OR namibia:ab,ti OR 'north macedonia':ab,ti OR panama:ab,ti OR paraguay:ab,ti OR peru:ab,ti OR romania:ab,ti OR russia:ab,ti OR 'russian federation':ab,ti OR serbia:ab,ti OR 'saint lucia':ab,ti OR 'saint vincent':ab,ti OR 'the grenadines':ab,ti OR suriname:ab,ti OR thailand:ab,ti OR tongo:ab,ti OR republic:ab,ti OR turkey:ab,ti OR turkmenistan:ab,ti OR tuvalu:ab,ti)

**Scopus**

( TITLE-ABS ( ( "severe acute respiratory syndrome coronavirus 2" OR "covid 19" OR ncov* OR covid* OR "sars cov 2" OR "sars-cov-2" OR "sars coronavirus 2" OR "severe acute respiratory syndrome cov 2" OR "wuhan coronavirus" OR "wuhan seafood market pneumonia virus" OR sars2 OR "2019-ncov" OR "hcov-19" OR "novel 2019 coronavirus" OR "2019 novel coronavirus*" OR "novel coronavirus 2019" OR "2019 novel human coronavirus*" OR "human coronavirus 2019" OR "coronavirus disease-19" OR "corona virus disease-19" OR "coronavirus disease 2019" OR "corona virus disease 2019" OR "2019 coronavirus disease" OR "novel coronavirus 2019*" OR "novel coronavirus disease 2019" OR "novel coronavirus infection 2019" OR "2019 corona virus disease" OR "new coronavirus*" OR "coronavirus outbreak" OR "coronavirus epidemic" OR "coronavirus pandemic" OR "pandemic of coronavirus" ) )

AND

TITLE-ABS ( ( "socioeconomics" OR socioeconomics OR "socioeconomic status" OR "socio-economic status" OR "socioeconomic position" OR "socio-economic position" OR "social determinants" OR "socioeconomic determinants" OR "socio-economic determinants" OR "social status" OR "social position" OR "socioeconomic class" OR "socio-economic class" OR "social class" OR "health disparities" OR "health inequalities" OR "social disparities" OR "social inequalities" OR "socioeconomic disparities" OR "socioeconomic inequalities" OR "socio-economic disparities" OR "socio-economic inequalities" OR "social gradient" OR "socioeconomic gradient" OR "socio-economic gradient" OR "socioeconomic differences" OR "socio-economic differences" OR "social differences" OR "health differences" OR "ses" OR "sep" OR income OR "income inequality" OR education OR "educational status" OR "educational inequality" OR employment OR "employment status" OR occupation OR occupational OR "wealth*" ) )

AND

TITLE-ABS ( ( prevalence OR "incidence" OR incidence OR "cumulative incidence" OR "incidence density rate" OR "incidence-density-rate" OR "person-time incidence rate" OR "attack rate" OR "attack-rate" OR "case rate" OR "case-rate" OR "case notification rate" OR "case-notification-rate" OR "infection risk" OR "infection-risk" OR "infection" OR infection OR "mortality" OR mortality OR "mortality-rate" OR "death rate" OR "death-rate" OR "death*" OR hospitalisation OR "hospitalisation rate" OR "hospitalisation-rate" OR "hospitalization" OR hospitalization OR "hospitalization rate" OR "hospitalization-rate" ) )

AND NOT

TITLE-ABS ( ( afghanistan OR "burkina faso" OR burundi OR "central african republic" OR chad OR eritrea OR ethiopia OR gambia OR guinea OR "guinea-bissau" OR "north korea" OR liberia OR madagascar OR malawi OR mali OR mozambique OR niger OR rwanda OR "sierra leone" OR somalia OR "south sudan" OR sudan OR "syrian arab republic" OR syria OR togo OR uganda OR yemen OR angola OR algeria OR bangladesh OR belize OR benin OR bhutan OR bolivia OR "capo verde" OR cambodia OR cameroon OR comoros OR congo OR "cote d`ivoire" OR djibouti OR egypt OR "el salvador" OR eswatini OR ghana OR haiti OR honduras OR indonesia OR iran OR kenya OR kiribati OR kyrgyzstan OR laos OR lesotho OR mauritania OR "federated states of micronesia" OR mongolia OR morocco OR myanmar OR nepal OR nicaragua OR nigeria OR pakistan OR "papua new guinea" OR philippines OR samoa OR "sao tome" OR principe OR senegal OR "solomon islands" OR "sri lanka" OR tanzania OR tajikistan OR "timor leste" OR tunisia OR ukraine OR uzbekistan OR vanuatu OR vietnam OR "gaza strip palestine" OR zambia OR zimbabwe OR albania OR "american samoa" OR argentina OR armenia OR azerbaijan OR belarus OR bosnia OR herzegovina OR botswana OR bulgaria OR colombia OR "costa rica" OR cuba OR dominica OR "dominican republic" OR "equatorial guinea" OR ecuador OR fiji OR gabon OR grenada OR guatemala OR guyana OR iraq OR jamaica OR jordan OR kazakhstan OR kosovo OR lebanon OR libya OR malaysia OR maldives OR "marshall islands" OR mauritius OR mexico OR moldova OR montenegro OR republic OR namibia OR "north macedonia" OR panama OR paraguay OR peru OR romania OR russia OR "russian federation" OR serbia OR "saint lucia" OR "saint vincent" OR "the grenadines" OR suriname OR thailand OR tongo OR turkey OR republic OR turkey OR turkmenistan OR tuvalu ) ) )

AND

( LIMIT-TO ( PUBYEAR , 2021 ) OR LIMIT-TO ( PUBYEAR , 2020 ) )

AND

( LIMIT-TO ( LANGUAGE , "English" ) OR LIMIT-TO ( LANGUAGE , "German" ) )
